# B cells in tumor metastasis: friend or foe?

**DOI:** 10.7150/ijbs.79482

**Published:** 2023-04-29

**Authors:** Yuqiu Xu, Yihao Mao, Yang Lv, Wentao Tang, Jianmin Xu

**Affiliations:** Department of General Surgery, Zhongshan Hospital, Fudan University, Shanghai, China

**Keywords:** B cell, tumor metastasis, mechanism, immunotherapy, metabolism

## Abstract

Metastasis is an important cause of cancer-related death. Immunotherapy may be an effective way to prevent and treat tumor metastasis in the future. Currently, many studies have focused on T cells, whereas fewer have focused on B cells and their subsets. B cells play an important role in tumor metastasis. They not only secrete antibodies and various cytokines but also function in antigen presentation to directly or indirectly participate in tumor immunity. Furthermore, B cells are involved in both inhibiting and promoting tumor metastasis, which demonstrates the complexity of B cells in tumor immunity. Moreover, different subgroups of B cells have distinct functions. The functions of B cells are also affected by the tumor microenvironment, and the metabolic homeostasis of B cells is also closely related to their function. In this review, we summarize the role of B cells in tumor metastasis, analyze the mechanisms of B cells, and discuss the current status and prospects of B cells in immunotherapy.

## 1. Introduction

The morbidity and mortality of patients with malignant tumors are increasing yearly, and tumor metastasis is the main cause of death. After tumor metastasis, conventional treatments such as surgery, radiotherapy and chemotherapy often have limited efficacy; therefore, immunotherapy provides great hope for patients. Most immunotherapies currently focus on T cells, such as the immune checkpoint inhibitor PD-1/PD-L1 1. However, not all patients with metastatic tumors benefit from immune checkpoint inhibitors. As an important part of adaptive immunity, B cells not only play an important role in humoral immunity but they are also critically involved in cellular immunity. However, the role of B cells in tumor metastasis is not clear. This paper summarizes and analyzes the role of B cells in regulating tumor metastasis and the current situation and prospects of B cells in the treatment of tumor metastasis.

## 2. Development and differentiation of B cells

B cells develop and differentiate from hematopoietic precursors in bone marrow. Functional rearrangements of heavy chain (μ chain) gene fragments allow pro-B cells to enter the pre-B-cell stage. Pre-B cells undergo functional rearrangement of light chains (κ and λ chains) and expression of IgM molecules to develop into immature B cells 2. Immature B cells leave the bone marrow after negative selection for central immunotolerance, migrate to the spleen, and differentiate into follicular B cells and marginal zone B cells 3. Immature B cells that routinely enter the circulation migrate to secondary lymphoid organs, such as the spleen, lymph nodes, and mucosal tissues, home to lymphoid follicles, form germinal centers (GCs), and develop into activated B cells through positive selection, class switching, and somatic hypermutation (SHM). Activated B cells can further differentiate into memory B cells or plasma cells.

B cells exhibit many phenotypes throughout the development and differentiation process. Naïve B cells, Mz-B cells, Fo-B cells and memory B cells enter the peripheral blood and tissue sites. These B cells play a role in antigen presentation and assist T-cell functions by expressing MHCII, CD80, CD86, CD69 and other surface markers. They can also secrete cytokines such as IL-2, IL-4 and IFN 4. Terminally differentiated plasma cells participate in humoral immunity by secreting IgG and other antibodies. During the process of differentiation, regulatory B cells (Bregs) can arise from any developmental stage. Bregs are characterized by producing immunosuppressive cytokines such as IL-10, IL-35 and TGF-β and/or expressing checkpoint ligands such as PD-1. Due to the lack of specific lineage markers, the phenotypes of Bregs are still unclear. Identified subsets of Bregs include CD19^+^CD24^Hi^CD27^+^ B10 cells, CD19^+^CD24^Hi^CD38^Hi^ immature B cells 5, and others, such as PD-1^Hi^CD5^Hi^CD27^+^CD38^Dim^ Bregs 6.

The tumor microenvironment (TME) is largely different from that of healthy tissues. By analyzing tumor tissues, several studies have described the importance of tumor infiltrating B cells (TIL-Bs) in the antitumor response. TIL-Bs reside in various structural zones of the TME, including organized tertiary lymphoid structures (TLSs) and less organized stromal infiltrates (nonfollicular aggregates of different immune cells), or directly infiltrate the tumor epithelium 7. Single-cell RNA sequencing has revealed that the TIL-Bs cover entire phenotypes throughout the developmental stages from naïve B cells to plasma cells, including *IL10*-expressing TIL-Bs 8, 9. Thus, TIL-Bs exhibit similar phenotypes as healthy tissues but have more heterogeneous subsets. This may be caused by the distinct evenness of B cell receptor (BCR) repertoires and different diversification of B-cell clones from SHM compared with nontumor tissues 10. In addition, considering the potential ability of TIL-Bs to recognize tumor-associated antigens (belonging to self-antigens), it is possible that tolerance disruption occurs during the development of TIL-Bs. TIL-Bs are the main force of local antitumor immunity, but their developmental process, subset markers and functions need further exploration.

## 3. Characteristics of B cells in metastatic tumor microenvironment

Malignant tumors are heterogenous, and the composition of the immune microenvironment of primary tumors and metastatic tumors exhibits significant differences 11, 12. Chen et al investigated the primary and metastatic TME of melanoma. They discovered that tumor-associated B cells of metastatic sites were present in stromal septa within the tumor and at the invasive tumor stroma margin, but those of primary sites exhibited a rather patchy, predominant paratumoral distribution at the invasive tumor-stroma margin. They also found that memory-like B cells were significantly decreased at distant metastatic sites compared to primary sites without metastasis but similar to primary sites with metastasis, while the infiltration of plasma cell-like cells was markedly higher in contrast with both kinds of primary sites 13. Similarly, Wu et al indicated that the proportion of AIM2^+^ memory B cells was reduced in colorectal liver metastases compared with adjacent colorectal samples 14.

The numbers and subsets of B cells in metastatic sites tend to be heterogeneous and are affected by various factors such as age and the surrounding TME. A higher age is associated with higher numbers of plasmablast-like cells 13. Olalekan et al. analyzed omental metastases from ovarian cancer and divided samples into 2 groups according to T-cell infiltration. The group with higher T-cell infiltration possessed interferon gamma (INFG)-expressing plasmablasts, but none of B-cell subsets in the other group expressed INFG 15. Additionally, not only immune cells but also chemokines of the surrounding TME have an impact on B cells. CXCL13 can drive IL-10-producing B cells to metastatic sites in experimental pulmonary metastatic models 16.

## 4. Association between B cells and prognosis of tumor metastasis

Studies of the tumor immune microenvironment have revealed that the prognosis of patients can be evaluated by the infiltration of immune cells into tumors and metastases 17, 18. The T-cell and B-cell infiltration density (TB score) was calculated by immunohistochemical labeling of tumor samples with CD8 and CD20. A higher TB score was associated with a better prognosis 19. High infiltration of B cells was closely associated with a better prognosis in a variety of tumor types, including breast, ovarian, renal cell, thyroid papillary and lung cancers 20-23. Furthermore, TIL-Bs were related to negative lymphatic invasion in gastric cancer, lymph node metastases in papillary thyroid carcinoma, and negative distant metastasis in colorectal cancer 23, 24. Elevated numbers of peripheral B cells could also help to improve the survival of locally advanced or metastatic pancreatic cancer 25. In addition, plasmablasts were found to be significantly increased in patients with metastatic melanoma, lung adenocarcinoma, and renal cell carcinoma without tumor progression for at least one year 26. This finding suggested that there was a persistent B-cell response in patients whose cancer has stopped progressing after antitumor therapy, and this may also be an important reason why tumor progression is suppressed.

However, some studies have not observed a positive role of B cells in survival. Bregs are usually associated with poor prognosis 5. For example, PD-L1^+^ Bregs are significantly increased in melanoma patients with bone metastasis 27. Increased leucine-tRNA-synthase-2 (LARS2)-expressing B cells (LARS B) indicated shortened survival in colorectal cancer patients 28. A meta-analysis of 69 studies including 19 cancers suggested that 50% of the studies reported a neutral or negative effect of TIL-Bs on prognosis 29. A phase I trial combined anti-CD20 antibody treatment with chemotherapy to treat recurrent or metastatic head and neck squamous-cell carcinoma and found that B-cell depletion was beneficial to disease-free duration 30.

The above results demonstrate the complex roles of B cells in tumor metastasis. The use of pan-B-cell markers such as CD19 and CD20 to predict metastasis and assess prognosis is not satisfactory. The expression of a single B-cell subtype to evaluate tumor metastasis may also not be sufficient. Most likely, the ratio of different B-cell subtypes can be a potential indicator, which requires a better understanding of B-cell effects on tumor metastasis and the corresponding mechanisms.

## 5. Inhibition of tumor metastasis by B cells and related mechanisms

### 5.1 High expression of BCRs promotes B cells to inhibit tumor metastasis

Activation of B cells is usually initiated by the binding of BCR to a specific antigen, which phosphorylates the residues of Igα/β heterodimer intracellular immune receptor tyrosine activation motifs (ITAMs), initiating the activation and signal transduction of SYK, BTK and other downstream molecules 31. Antigen exposure leads to the recombination of BCRs and the cytoskeleton, and BCRs aggregate into BCR microclusters, thus promoting the activation of B cells. By analyzing the mRNA sequencing of B cells, Michael et al. 20 found that in some tumor subtypes, such as basal-like breast cancer, HER2-enriched breast cancer and immunoreactive ovarian cancer, which might be candidates for identifying productive antitumor B-cell responses, the expression of B-cell-associated genes and BCR segments was significantly higher. The expression of some BCR segments, such as IgHV and IgKC, was positively associated with improved metastasis-free survival of these tumor subtypes. Moreover, the highly expressed BCR segments presented decreased BCR diversity, and the mutation patterns were in accordance with BCR SMH. The presence of SHM characteristics was suggestive of the presence of antigen-experienced B cells, which were potentially against tumor antigens. IgKC gene expression was also associated with better prognosis of colorectal cancer and non-small cell lung cancer 32. Therefore, it is likely that decreased diversity of highly expressed BCR gene segments may serve to characterize the types of B cells that function to inhibit tumor metastasis.

The signal transduction and function of BCRs depend on the normal expression and function of various downstream molecules, such as BLNK and BTK 33, 34, as well as the synergistic action of the cell membrane, actin skeleton and related regulatory proteins, such as WAS protein 35, to perform their subsequent transcriptional conduction function. Dysregulated expression of various downstream molecules and regulatory proteins will also affect the ability of B cells to inhibit tumor metastasis. For example, the missing-in-metastasis/metastasis suppressor 1 (MIM/MTSS1) gene is closely related to the normal function of BCR and B cells 36. MIM is a membrane and actin skeleton regulatory protein that is highly expressed in the spleen, especially in B cells 37. The loss of MIM not only affects the binding of BCR to antigens but also affects the expression of B-cell antibodies, thus inhibiting the function of B cells.

In summary, the inhibition of tumor metastasis by B cells may be related to the diversity, expression and signal transduction of BCRs.

### 5.2 B cells assist other immune cells in suppressing tumor metastasis

One of the important functions of B cells is to secrete a variety of immunoglobulins, which play an antitumor immune role through the antibody-dependent cell-mediated cytotoxicity (ADCC) pathway, antibody-dependent cell-mediated phagocytosis (ADCP) or the complement-dependent cytotoxicity (CDC) pathway 38. In addition, B cells can present antigens and assist many types of immune cells.

B cells can enhance the ability of dendritic cells (DCs) to recruit, differentiate and present antigens. In a study of high-grade serous ovarian omental metastasis, memory B cells recruited DCs to the site of tumor metastasis by secreting CXCL8, enhancing their antigen-presentation role 39. B cells also contribute to the maturation of DCs 40, thus inducing T cells to have a stronger cell killing ability 41.

B cells activate natural killer (NK) cells by secreting IL-12. Studies on NK-sensitive metastatic lung cancer found that when lymphocyte infiltration at the lung metastasis site was reduced by a stress test, the reduction in B cells was particularly obvious, and the secretion of IL-12 by B cells was reduced, which weakened the activation and function of NK cells and then led to an increase in lung metastasis 21. After neutralizing B cells with antibodies, tumor metastasis increased, and the effect was similar to that of neutralizing NK cells 42, 43. This result reveals the function of B cells in promoting NK cells and inhibiting tumor metastasis.

Furthermore, B cells can promote the role of CD4^+^ and CD8^+^ T cells. In a B-cell-deficient mouse model of lung metastasis from melanoma, T cells that secrete IFNγ and TNFα are reduced, and the proliferation of tumor-specific CD8^+^ T cells is significantly reduced 44. Adoptive transfer of activated B cells into a model of colorectal cancer can reduce liver metastasis and increase liver infiltration of cytotoxic granzyme B (GzmB) ^+^ T cells 45. Recent studies have shown that after introducing conjugated B-cell- and CD4 T-cell-recognized neoantigens into a lung adenocarcinoma mouse model, B cells induce tumor antigen-specific follicle-assisted CD4^+^ T cells (TFHs) to secrete IL-21 and promote the production of GzmB by tumor-infiltrating CD8^+^ T cells 46. In this process, B cells play the role of antigen-presenting cells, which contributes to the antitumor immunity of T cells.

In conclusion, B cells can not only secrete antibodies to exert immune functions but also inhibit tumor metastasis by assisting DCs, NK cells and T cells.

### 5.3 Direct killing ability of B cells

In addition to assisting other immune cells, an increasing number of studies have indicated that the direct killing ability of B cells also plays an important role in the immune response to tumor metastasis. In a study of spontaneous lung metastasis of breast cancer, IL10-negative effector B cells overexpressed FasL and played a direct killing role in inhibiting the progression of metastasis through the Fas-FasL pathway. The expression of Fas in tumor cells was also significantly increased *in vitro*, indicating that this type of B-cell had high efficiency 47. By studying a model of spontaneous lung metastasis induced by the tumor cell line 4T1, investigators also discovered that CXCR4 was expressed on tumor-draining lymph node B cells, which could attract CXCL12-producing 4T1 cells and kill tumor cells by the perforin pathway 48. B cells could also inhibit tumor metastasis by secreting IFNγ and promoting the dissolution of metastases 49.

Increasing evidence shows that GzmB-producing B cells may possess cytotoxic potential. In patients with invasive breast ductal carcinoma, GzmB^+^ B cells are markedly decreased in metastatic lymph nodes compared with nonmetastatic lymph nodes 50. B-cell-origin GzmB is transferred from B cells to HeLa cells and results in apoptotic shrinkage of HeLa cells 51. For the production of GzmB by B cells, BCR stimulation is essential, which may imply that GzmB production is antigen-specific 50. BCRs also play an important role in class switch recombination (CSR) of B cells. After CSR, B cells switch from expressing IgM to other classes of antibodies such as IgG and IgA 52. These antibodies display some new functions for inhibiting tumor progression directly. For example, TIL-B-origin IgG can promote degradation of the tumor protein by antibody-dependent intracellular neutralization, and TIL-B-origin IgA impairs oncogenic signaling by transcytosis 53, 54.

### 5.4 B-cell and tertiary lymphoid structures (TLSs)

TLSs are ectopic lymphoid organs that exist in various nonlymphoid tissues, such as primary and metastatic tumors, where TIL-Bs predominantly reside 55. TLSs are associated with better patient outcomes in breast, lung, melanoma, and ovarian cancers 39, 56, 57. CXCR5+ TIL-Bs, which are recruited by CXCL13, play an important role in TLS formation and maturation. Without TLSs, the function of TIL-Bs may be weakened and become suppressive 58. In the TLSs of melanoma skin metastasis, TIL-Bs present clonal amplification, rearranged immunoglobulin genes, somatic hypermutation and isotype switching. In the TLSs of omental metastasis from high-grade serous ovarian cancer, memory B cells also show a high degree of clonality and somatic hypermutation rate and produce chemokines that attract DCs, T cells and NK cells 59. TIL-Bs in TLSs can also impair the impact of Tregs on lung cancer 60. In addition, the markers of B cells in tumor tissues are significantly increased in patients with metastatic melanoma and renal cell carcinoma who have a good response to immune checkpoint inhibitors. Immunofluorescence assays have shown that the number and diversity of receptors for these TIL-Bs is increased significantly 56, 57. Further investigation reveals that switched memory B cells are the main subtypes of increased TIL-Bs, and they may also contribute to an effective T-cell response after immune checkpoint blockade 56. Switched memory B cells are also enriched in TLSs of pancreatic ductal adenocarcinoma and associated with longer survival 61. In brief, B cells infiltrated at both primary and metastatic sites of tumors mainly reside in TLSs, which provide sites for the activation and further differentiation of B cells into antitumor immune cells. The role of TIL-Bs in inhibiting tumor metastasis is closely related to TLSs.

## 6. Promotion of tumor metastasis by B cells and related mechanisms

### 6.1 Regulatory B cells promote tumor metastasis

During the process of development and differentiation, B cells are regulated by a variety of immune cells, tumor cells, cytokines and chemokines in the surrounding immune microenvironment and eventually express different cell surface markers, secrete different molecules and play different roles. Therefore, many studies have shown that B cells promote tumor progression. These B cells are usually classified as regulatory B cells (Bregs).

Bregs are immune regulatory cells that can maintain immune tolerance, inhibit autoimmune responses, inflammatory responses and antitumor immunity, secrete some anti-inflammatory cytokines such as IL-10 and IL-35, and express some inhibitory molecules such as PD-L1^62^-^65^. Bregs have been proven to promote tumor metastasis in a variety of cancers, such as breast, lung, colorectal, esophageal, pancreatic and skin cancers 65-68. Studies have shown that the depletion of B cells by administering anti-IgM serum or anti-CD20 antibody can effectively inhibit tumor metastasis 69, 70. CD5^+^ Bregs inhibit antitumor immunity by activating STAT3 and secreting the immunosuppressive factors IL-10 and IL-35^71^. CD5^+^ Bregs can also express CCR6, which interacts with tumor-derived CCL20 and promotes the progression of hepatocellular carcinoma 72. Bregs also inhibit the antitumor effects of other immune cells. Bregs induce TGF-β activation by dendritic cells through the production of enzyme-stimulating thrombospondin-1 (TSP-1) to exert immunosuppressive effects 73. Bregs can inhibit the antitumor immunity of T cells in a variety of ways. 1) Although the GzmB-producing B cells mentioned above can kill tumor cells, GzmB^+^ Bregs impede antitumor immunity 51. GzmB produced by Bregs can degrade TCR on CD4^+^ T cells 74. It is likely that tumor subtype, activation status of other immune cells and molecular expression on B cells jointly determine the role of GzmB^+^ B cells in antitumor immunity. 2) Bregs induce T-cell differentiation into Tregs and promote tumor metastasis, and studies have shown that breast cancer patients with high expression of Tregs and Bregs have a significantly shorter metastasis-free survival compared to those with only high Treg expression 75. 3) Bregs expressing PD-L1 inhibit proinflammatory T-cell function by binding to PD-1^63^. 4) Bregs expressing Fas-L induce target cell death by binding to Fas to inhibit proinflammatory T-cell differentiation 76.

Bregs can influence other antitumor cells by constructing an inhibitory immune environment and they can also directly interact with tumor cells to promote tumor metastasis. Because of lacking specific marker like FOXP3, it is hard to identify Bregs. The subsets of Bregs are intricate, which makes it difficult to treat them as targets. However, it is true that TME influence their differentiation. TIL-Bs in mature TLS tend to promote anti-tumor immunity, while B cells in immature TLS tend to become a regulatory phenotype. Remodeling TME such as expanding mature TLS is probably a potential method.

### 6.2 Tumor cells induce B cells to promote tumor metastasis

Tumor cells can alter the phenotype and function of B cells to achieve immune escape and a favorable immune microenvironment for metastasis.

B cells generally differentiate into immature B cells in the bone marrow and then enter the peripheral circulation, homing to secondary lymphoid organs for subsequent differentiation. However, studies on ovarian and breast cancer have found that earlier-stage B cells, such as pre-B-like B cells, exist in the peripheral circulation. It was found that thymic stromal lymphopoietin (TSLP) secreted by tumor cells downregulated the expression of CXCR4 and VLA4 in B-cell precursors, enhanced their mobility, enabled them to migrate out of bone marrow, and induced the transformation of B-cell precursors into Bregs to promote tumor metastasis 77. Further exploration revealed that cancer also induced the differentiation of these B-cell precursors into macrophage-like cells (B-MFs). B-MF not only suppressed the proliferation of T cells, but also promoted the differentiation of Tregs 78.

As an important component of lymph nodes, B cells also play an important role in lymphangiogenesis, suggesting that B cells may promote lymphatic metastasis of tumors 79. The TME may influence the differentiation of B cells, resulting in the formation of tumor-educated B cells. In a model of spontaneous lymph node metastasis of breast cancer, these unique B cells accumulated in tumor-draining lymph nodes and produced a large amount of pathologic IgG immunoglobulin, which activated the NF-κB pathway by binding to the tumor cell surface HSPA4 protein. Furthermore, the CXCR4/SDF1α axis promoted tumor lymph node metastasis 80. Tumor-educated B cells were also dramatically recruited in renal cell carcinoma to promote metastasis by secreting IL-1β and inducing HIF-2α/Notch1/MMP9 signaling 81.

In the primary site, tumor cells induce B cells to promote the migration of tumor cells. In the metastatic site, tumor cells induce B cells to create suitable “soil” for tumor colonization. Whether there are common sites on the surface of B-cell that can interact with different types of tumors, and whether the mature TLSs can protect B cells from the influence of tumor cells are worth further exploration.

### 6.3 Disorder of B-cell metabolism

To meet the balance of tumor energy supply, continuous macromolecule synthesis and oxidation-reduction reactions, tumor cell metabolism undergoes significant changes during tumorigenesis, progression and metastasis 82. In addition, the metabolism of immune cells in the TME is also affected. Tumor cells and immune cells are concentrated in the same nutrient- and oxygen-limited microenvironment, which forces them to compete with each other for limited nutrients to meet their own needs 83, 84. Tumor cells need to divide and migrate, while immune cells need to be activated and performed their functions. However, tumor cells with high invasive ability often have the upper hand in this competition. During tumor progression and metastasis, the metabolism of immune cells is dysregulated, and their function is disrupted. At present, there are many studies on the metabolism of T cells 85, but little is known about the metabolism of B cells.

When BCRs, Toll- like receptor (TLR) or cell surface costimulatory molecules such as CD40 are activated, B cells proliferate rapidly, and the biomass energy in B cells is upregulated rapidly. BCRs and TLRs significantly upregulate glucose and amino acid transport. Glucose mainly enters the pentose phosphate pathway for metabolism 86, 87. In addition to the large amount of energy required for B-cell activation, plasma cells also need a large number of biosynthetic precursor molecules and carbon sources to produce energy during antibody production 88. Both short-lived and long-lived plasma cells require glucose uptake for glycosylation of proteins 89. Long-lived plasma cells maintain cell survival by absorbing glucose, converting it into pyruvate and entering the tricarboxylic acid cycle (TAC)^89^. Plasma cells also need to consume amino acids to perform their functions 90. In addition, autophagy has been found to play an important role in plasma cell metabolic homeostasis and memory B-cell survival 91. Regardless of whether plasma cells or memory B cells are the main force in antitumor immunity, metabolic disorders will seriously affect their function or even survival.

Both the tricarboxylic acid cycle and pentose phosphate pathways are oxygen-dependent metabolic pathways, but the TME is often hypoxic. At this point, B cells produce hypoxia-inducible factor (HIF-1α) to regulate glycolysis 90. In animal models, HIF-1α regulates IL-10 transcription and induces the proliferation of IL-10-producing CD1d^hi^ CD5^+^ B cells (B10) 92. Therefore, a hypoxic environment may be the driving factor of Breg formation, and preventing tumor progression by an oxygen-sensing mechanism in tumors may also inhibit Breg differentiation and improve patient prognosis. Recent studies have also found that γ-aminobutyric acid (GABA), a metabolite of glutamate in B cells, promotes the emergence of anti-inflammatory macrophages and drives the antitumor response of cytotoxic T cells 93. This significant discovery also provides a new metabolic target for tumor immunotherapy.

It seems that the differentiation of B cells into antibody-producing B cell subsets or regulatory B cell subsets is largely related to the selection of bioenergetic pathways. Changing the anoxic environment outside B cells or changing the oxygen sensing pathway inside B cells may be beneficial to improve the antitumor function of B cells. In addition, more studies are required to clarify the relationships between metabolic pathways and the presentation of distinct markers or the production of different cytokines and antibodies, so as to provide metabolic targets of B cells.

## 7. Therapeutic manipulation of B cells to inhibit tumor metastasis

Regarding immunotherapy for the treatment of tumor metastasis, T cells and DCs have been the main focus, with the treatments of PD-1/PD-L1 immune checkpoint inhibitors and DC tumor vaccines. B cells, as an important component of tumor immunity, have good prospects for further exploration and development into treatments.

### 7.1 Cytokine and chemokine-related therapies

Cytokines and chemokines play important roles in the proliferation, differentiation and migration of B cells. Intratumor injection of cytokines, such as IL-12 and B-cell-activating factor (BAFF), can help improve the prognosis of tumor metastasis. IL-12 can activate B cells and induce immunoglobulin production and IFNγ secretion 94. BAFF is a B-cell activating cytokine. Higher expression of BAFF is associated with improved 5-year overall survival of metastatic melanoma. BAFF can upregulate B-cell costimulatory molecules and enhance B-cell antigen-presentation to CD4^+^ Th cells 95.

Inducing B-cell migration and the formation of TLSs are also beneficial to antitumor metastasis therapy. The cytokine IL-36γ is beneficial to TLS formation, and is also correlated with the increased density of TIL-Bs in TLSs 96. The CCL19, CCL21/CCR7 and CXCL13/CXCR5 axes are important chemotactic migration pathways that induce B-cell migration into the tumor immune microenvironment 97, 98. These chemokines are also required for the formation of TLSs 99. However, the function of the CCL19, CCL21/CCR7 and CXCL13/CXCR5 axes in tumor immunity is complicated. Moreover, CCR7 and CXCR5 are also essential for the migration of cancer cells 100. Studies have indicated that the paracrine signals of these axes are beneficial to B-cell migration to the TME and the development of TLSs, while the autocrine signal of these axes may promote the proliferation and metastasis of cancer cells 38. In addition, these axes can also promote the migration of immunosuppressive B-cell subsets such as Bregs 101. How to regulate these chemokine pathways is a challenging issue. Pylayeva-Gupta et al. revealed that CXCL13, secreted by fibroblasts, induced the migration of IL-35-producing Bregs 68. However, in DC-based immunotherapy, injection of CCL21 can promote tumor regression 102. In addition, CXCR5 is particularly prominent in pancreatic carcinoma, but its expression is low in CT26 colon carcinoma 103. These findings indicate that the function of these axes related to the “pro- and anti-tumor” roles of B cells is determined by different TMEs including secretion modes, receptor-expressing cells and types of cancer. Different strategies need to be adopted according to the heterogeneous TME to inhibit the function of Bregs and enhance the function of antitumor B cells.

### 7.2 Chemotherapy and immune checkpoint inhibitors

Chemotherapy can increase B-cell infiltration. B cells with a strong memory response infiltrating lymphoid structures in high-grade serous ovarian cancer metastases are associated with a better prognosis, and chemotherapy can enhance the response 39. Neoadjuvant chemotherapy and Doxorubicin can increase the numbers of B cells in breast cancer, ovarian cancer and bladder cancer 104-106. Chemotherapy combined with macrophage inhibition helps increase B-cell infiltration and causes tumor regression in triple-negative breast cancer 107.

As mentioned above, an increase in B cells can contribute to an effective T-cell response after immune checkpoint blockade 56. Interestingly, the immune checkpoint inhibitor (ICI) anti-PD1 can not only improve the function of T cell, but also enhance B-cell activation capacity 108. PD-L1^+^ B cells are targets of ICIs as well. However, B-cell-specific immune checkpoints remain unclear. CXCL13 deficiency is associated with reduced IL-10-producing B cells in metastatic sites, as well as improved efficacy of chemotherapy and anti-PD1 16. CXCL13 is a promising immune checkpoint of Bregs. However, the function of CXCL13 is complex, as mentioned above. Further exploration of B-cell-specific immune checkpoints is still needed to synergize B-cell and T-cell antitumor effects by combining checkpoint inhibitors.

### 7.3 Adoptive transfer of B cells

A sufficient level of activated B cells is significant for antitumor immunity. Adoptive transfer can be performed after activation of B cells *in vitro*, mimicking a T-cell vaccine. Several animal experiments have shown that the adoptive transfer of activated B cells *in vitro* into animals with tumors can effectively inhibit tumor progression and metastasis 45, 49, 109. B cells can be activated by CD40 *in vitro*, which not only has the ability to present antigens like DC vaccines but can also assist T-cell function. The addition of IL-2 can further enhance the direct killing ability of adoptively transferred B cells 48. Considering that anti-PD1 can enhance B-cell activation capacity, it may provide the possibility of combining immune checkpoint inhibitors with adoptively transferred B cells to inhibit tumor metastasis.

### 7.4 Extracellular vesicles

An increasing number of studies have focused on extracellular vesicles (EVs) because EVs are cell-free, have low toxicity, are more effective and can be stored for a longer time 110. B cells can release MHC class II antigen-presenting EVs, which can elicit antigen-specific CD4^+^ T-cell responses 111 and enhance the antigen-presenting ability of follicular DCs by decorating them with MHC-II at their plasma membranes 112. EVs secreted by T cells and DCs can in turn promote the proliferation and differentiation of B cells 112, and EVs from DCs also induce increased germinal center B-cell proportions 113. Therefore, EVs from B cells may be used as adjuvants for DC vaccines or T-cell adoptive cell transfer, and EVs from DCs and T cells may also be used as adjuvants for adoptively transferred B cells.

### 7.5 Natural compounds

Some natural compounds can also enhance the function of B cells. Resveratrol, a plant-derived polyphenol, can inhibit lung metastasis in a mouse model by preventing the generation and function of tumor-evoked regulatory B cells 114. *Ganoderma lucidum* polysaccharides can inhibit tumor growth and metastasis by inducing B cells to activate an IgM-mediated cytotoxic pathway 115. Botanical drugs P_4_N, a derivative of the plant lignan nordihydroguaiaretic acid, can increase the proliferation of TIL-Bs. P_4_N can also increase the production of antibodies via the leukotriene A4 hydrolase (LTA4H)/activin A/BAFF pathway 116. Dose control presents a challenge in the application of these natural compounds. The doses used should be effective while minimizing toxicity and side effects, which needs to be evaluated in more animal and clinical trials.

### 7.6 Regulation of B-cell metabolism

Both intracellular metabolic pathways and extracellular metabolites can impact the function of B cells. It is discovered that the production of IL-10 in B cells is associated with cholesterol metabolism. Inhibition of cholesterol metabolism by using atorvastatin can suppress the production of IL-10 and impede the ability of B cells suppressing effector T cell differentiation 117.

TGF-β1-expressing LARS B cells, a kind of immunoregulatory B cell, show a leucine nutrient preference. A leucine-dieting scheme can inhibit LARS B cells 28. Several studies of autoimmune diseases have demonstrated that Gut-microbiota-derived metabolites, such as short chain fatty acid (SCFA), can activate Bregs and inhibit germinal center B-cell and plasmablast differentiation 118. Dietary fiber supplementary in healthy individuals is related to increased B10 cells 119. In the occurrence of gastrointestinal tumors, how the metabolites of microbiome affect B-cell metabolism needs further study. These results indicate that diet adjustment may be beneficial to metabolic regulation, especially for gastrointestinal tumors.

## 8. Summary

**Figure 1:**
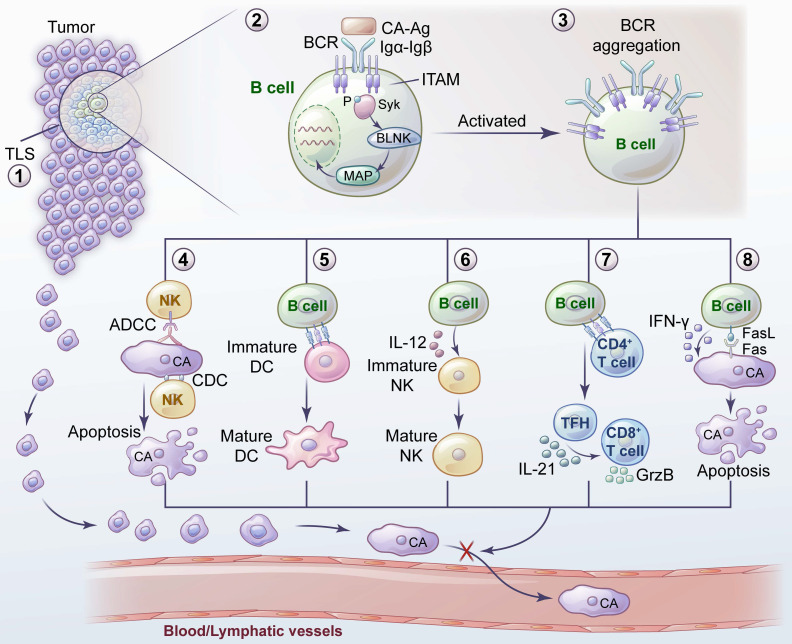
**Inhibition of tumor metastasis by B cells and related mechanisms.** 1) TLS provides sites for the activation and differentiation of B cells. 2) The combination of BCR and tumor antigens initiates the activation and signal transduction of downstream molecules. 3) Antigen exposure leads to BCR aggregation into BCR microclusters, thus promoting the activation and function of B cells. 4) Immunoglobulins secreted by B cells inhibit tumor metastasis through ADCC, ADCP and CDC. 5) B cells contribute to the maturation and recruitment of DCs at the site of tumor metastasis. 6) B cells facilitate the maturation of NK cells by secreting IL-12 to inhibit tumor metastasis. 7) B cells induce tumor-specific TFHs to secrete IL-21 and thus promote the production of GzmB by tumor-infiltrating CD8^+^ T cells. 8) Direct killing ability of B cells inhibits tumor metastasis.

**Figure 2:**
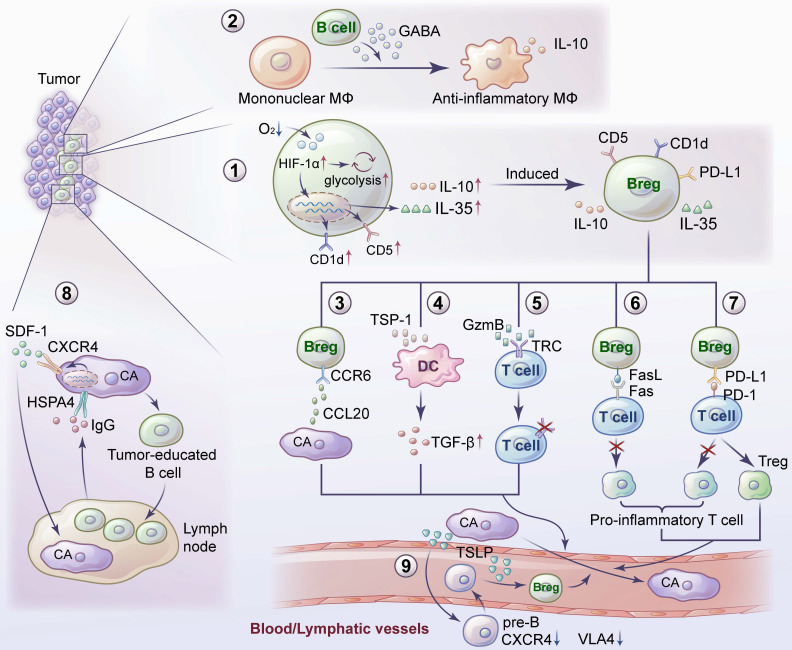
**Promotion of tumor metastasis by B cells and their related mechanisms.** 1) A hypoxic TME leads to an increase in HIF-1α in B cells, which induces the proliferation of IL-10- and IL-35- producing Bregs. 2) GABA, secreted by B cells, promotes the emergence of anti-inflammatory macrophages. 3) CCR6^+^ Bregs facilitate metastasis by interacting with tumor-derived CCL20. 4) Bregs induce TGF-β activation of DCs by producing TSP-1 to promote tumor metastasis. 5) GzmB secreted by Bregs degrades TCR on CD4^+^ T cells. 6) Bregs inhibit proinflammatory T-cell differentiation through the Fas-FasL pathway. 7) Bregs induce T-cell differentiation into Tregs and promote tumor metastasis by expressing PD-L1. 8) Tumor-educated B cells promote lymph node metastasis by secreting pathologic IgG, which activates the CXCR4/SDF1α axis by binding to tumor expressing HSPA4. 9) TSLP produced by tumor cells downregulates the CXCR4 and VLA4 expression in pre-B cells. TSLP enables pre-B cells to migrate, and induces them to transform into Bregs.

B cells are an important part of adaptive immunity. Compared with research on T cells, research on B cells is relatively scarce. B cells interact with tumor antigens through BCRs to initiate downstream signal transduction, enabling B-cell activation. Some B-cell subsets play an important role in inhibiting tumor metastasis (Figure 1), which may be due to the high expression of BCRs, assisting other antitumor immune cells, directly killing tumor cells, and activating TLSs. Some B-cell subsets play an important role in promoting tumor metastasis (Figure 2). These subsets may be Bregs expressing or secreting inhibitory factors such as IL-10 to establish an immune environment promoting tumor metastasis or other B-cell subsets that have not been clearly classified that promote tumor metastasis.

Because B cells play such a complex and diverse role in tumor metastasis, immunotherapy research on B cells is of great significance. Enhancing the function of B cells that inhibit tumor metastasis is feasible. However, the current research in this area is limited, so there are contradictions in the use of these two opposite therapeutic directions. One focus of future research is how to make B-cell immunotherapy more accurate to solve the heterogeneity of different B-cell subsets in different TMEs. Further exploration of B-cell markers as therapeutic targets and more efficient construction of mature TLSs may be beneficial. Another focus is how to better combine the immunotherapies of T cells with B cells. The combination of cellular and humoral immunity will be helpful to improve the efficiency of immunotherapy. Looking for B-cell immunity checkpoints, or adoptive transfer of both T cells and B cells needs further basic and clinical studies. In addition, recent advances have indicated that B-cell metabolism changes lead to changes in the differentiation direction and immune function of B cells. What are the changes occurring in B-cell metabolism in the TME? How do these changes relate to the metastasis of tumor cells? What are the differences in B-cell metabolic characteristics between primary and metastatic tumors? These questions require further investigation and also point to the possibility of regulating B-cell metabolism as an immunotherapy strategy. Overall, B-cell immunotherapy has great potential and promising prospects for preventing and treating tumor metastasis.
